# Assessing the Real-Time Mental Health Challenges of COVID-19 in Individuals With Serious Mental Illnesses: Protocol for a Quantitative Study

**DOI:** 10.2196/19203

**Published:** 2020-05-22

**Authors:** Raeanne Cristine Moore, Colin Andrew Depp, Philip D Harvey, Amy E Pinkham

**Affiliations:** 1 University of California San Diego San Diego, CA United States; 2 VA San Diego Healthcare System San Diego, CA United States; 3 University of Miami Miller School of Medicine Miami, FL United States; 4 Bruce W Carter VA Medical Center Miami, FL United States; 5 The University of Texas at Dallas Richardson, TX United States; 6 University of Texas Southwestern Medical School Dallas, TX United States

**Keywords:** mental disorders, technology, telemedicine, pandemic, psychology, stress, social distancing, coping, COVID-19, public health

## Abstract

**Background:**

The outbreak of coronavirus disease 2019 (COVID-19) has caused significant stress and mental health problems among the general public. However, persons at greatest risk for poor mental health outcomes, such as people with serious mental illness, have been largely overlooked.

**Objective:**

This paper presents the protocol for a study that aims to examine the mental health impact of COVID-19 and social distancing behaviors in people with serious mental illness and the behaviors undertaken to prevent COVID-19 infection in this group.

**Methods:**

Participants will include individuals with serious mental illness (eg, schizophrenia, bipolar disorder) and nonpsychiatric control participants who are currently participating in or have previously participated in several ongoing parent observational studies. Data will be collected from April 2020 through August 2020. Participants will complete phone interviews at 2 time points to assess their current emotional functioning and discuss the measures they have taken to prevent COVID-19 infection. Baseline (pre-COVID-19) mental health, sampled by ecological momentary assessment over an extended period, will be compared with current mental health, also sampled by ecological momentary assessment over an extended period. Demographic, cognitive, and psychosocial factors at baseline will be used to examine risk and resilience to current mental health and coping.

**Results:**

The inclusion of participants for the first round of telephone assessments started on April 3, 2020 and will be completed by May 31, 2020. As of April 30, 2020, 101 individuals had completed these first-round assessments. The second round of telephone assessments will likely occur between June 1, 2020, and August 31, 2020. Study results will be published in peer-reviewed scientific journals.

**Conclusions:**

Our findings will have broad implications for understanding the psychological consequences of COVID-19 among vulnerable persons with serious mental illness and will provide the opportunity to identify targets to reduce negative outcomes in the future. We also hope our efforts will provide a roadmap and resources for other researchers who would like to implement a similar approach.

**International Registered Report Identifier (IRRID):**

DERR1-10.2196/19203

## Introduction

Coronavirus disease 2019 (COVID-19) has created a global pandemic and disrupted our society and daily lives. Americans have been forced to separate from their workplaces and their friends, engage in previously foreign behaviors including “social distancing” and “sheltering in place,” and unemployment rates have jumped to unprecedented highs. The media have mainly focused on the psychological effects of COVID-19 in the general public, largely overlooking its impact on the most vulnerable groups in our society. The aim of this paper is to present the protocol for a study that will examine the effect of the pandemic on people with serious mental illness (SMI).

A recently published review in Lancet Psychiatry outlined the heightened risk of COVID-19 transmission among people with mental health disorders [1]. In addition to increased risk for infection, people with mental health disorders, and particularly those with SMI, could experience greater susceptibility to emotional responses to the pandemic, such as fear, anxiety, stress, depression, as well as risk of relapse or worsening of positive (eg, paranoia, hallucinations) and negative (eg, anhedonia, apathy) psychotic symptoms. This could be due to several reasons, including higher vulnerability to stress compared to the general population [2,3], reduced access to resources to permit ongoing mental health treatment and services [4,5], greater job or food insecurity [6,7], and additional restrictions in existing congregated situations such as group homes [8]. Social isolation or distancing may be less discrepant from daily living in some people with SMI than the general population. Moreover, some people with SMI may be less engaged in social networks and standard news media. Thus, the hypothesis of reduced subjective stress compared to the population in general needs to be considered.

On the other hand, health messages and awareness of the crisis are quite likely to not be well disseminated to people with SMI, creating a public health risk for individuals with SMI and others with whom they may have contact. An additional issue is challenges in the ability to understand and comply with complex directives and precautionary measures. People with SMI represent about 2%-3% of the population, and COVID-19 may result in collective increases in symptom severity, which, in turn, could result in expansive increases in mortality, emergency care utilization, and distress. Thus, it is critical to understand the influences of the COVID-19 pandemic on people with SMI. Further, our previous research has suggested that people with SMI are particularly challenged in self-assessments of both their emotional states and of their ability to engage in productive behaviors targeting their everyday functioning and self-management [9,10].

Our research team has two ongoing studies centered around Strategy 3.1 of the National Institute of Mental Health’s (NIMH) strategic plan to “identify and validate new targets for treatment development that underlie disease mechanisms” [11]. Both studies are multisite collaborations between the University of Texas at Dallas, University of California San Diego, and the University of Miami. Study 1 (principal investigator [PI]: author AEP, R01MH112620) assesses the construct of introspective accuracy, or the ability to correctly judge one’s own skills and abilities. The goals of this study are (1) to learn how impaired introspective accuracy in individuals with serious mental illness contributes to difficulties in real-world functioning, (2) to understand how introspective accuracy differs from other types of self-awareness, and (3) to discover how clinical symptoms affect the amount and direction of introspective accuracy impairments among outpatients with serious mental illness. To date, 189 participants aged 18-60 years have completed the study protocol (101 with schizophrenia or schizoaffective disorder, 72 with bipolar disorder, 16 controls; see Table 1 for baseline demographic and clinical characteristics of the sample), which includes an in-person, lab-based assessment followed by 30 days of at-home symptom tracking and cognitive testing via smartphone-based ecological momentary assessment (EMA). The goals of study 2 (PI: author CAD, R01MH116902) are to understand, over a 1-year period, how cognitive biases in the ways that outpatients with psychotic disorders (eg, schizophrenia, bipolar disorder with psychosis) perceive other people impact suicidal ideation and behavior. Ninety-seven participants aged 18-65 years have completed baseline assessments (38 with schizophrenia, 41 with schizoaffective disorder, 16 with bipolar disorder with psychosis, 2 with major depressive disorder and psychosis; see Table 2 for baseline demographic and clinical characteristics of the sample). Similar to study 1, the baseline assessments for study 2 include an in-person, lab-based assessment followed by 10 days of in-the-moment reports of symptoms and performance-based social cognition assessments via smartphone-based EMA.

**Table 1:** Participant demographic and clinical characteristics from parent study 1.

Characteristic		Patients (n=196)		Controls (n=16)	
Sex (male), n (%)		88 (45)		11 (69)	
**Race, n (%)**					
	Caucasian		79 (40)		10 (63)
	African American		82 (42)		4 (25)
	Native American		3 (2)		0 (0)
	Asian		6 (3)		1 (6)
	Native Hawaiian/Pacific Islander		2 (1)		0 ()
	Other		24 (12)		1 (6)
**Ethnicity, n (%)**					
	Hispanic		51 (26)		3 (19)
	Non-Hispanic		145 (74)		13 (81)
**Diagnosis, n (%)**					
	Schizophrenia		60 (31)		N/A^a^
	Schizoaffective disorder		52 (27)		N/A
	Bipolar disorder (with psychotic features)		45 (23)		N/A
	Bipolar disorder (without psychotic features)		38 (19)		N/A
**Employment status^b^, n (%)**					
	Employed, full time		22 (11)		13 (81)
	Employed, part time		25 (13)		1 (6)
	Unemployed		29 (15)		1 (6)
	Stay-at-home parent		2 (1)		0 (0)
	Part-time student		5 (3)		0 (0)
	Full-time student		6 (3)		2 (13)
	Receiving disability		96 (49)		0 (0)
	Receiving disability, part-time work		13 (7)		0 (0)
	Retired		6 (3)		0 (0)
**Residential status^c^, n (%)**					
	Independent, financially responsible		136 (69)		16 (100)
	Independent, not financially responsible		38 (19)		0 (0)
	Residential facility, unsupervised		8 (4)		0 (0)
	Residential facility, supervised		13 (7)		0 (0)
Age (years), mean (SD)		41.30 (10.97)		35.56 (9.06)	
Education (years), mean (SD)		13.30 (2.57)		15.13 (1.09)	
Maternal education (years)^d^, mean (SD)		13.11 (3.57)		13.75 (3.97)	
Paternal education (years)^e^, mean (SD)		13.59 (3.74)		14.69 (2.56)	
**Positive and Negative Syndrome Scale, mean (SD)**					
	Positive total		15.64 (5.08)		N/A
	Negative total		12.29 (3.91)		N/A
	General total		30.14 (7.03)		N/A
Montgomery-Asberg Depression Rating Scale total, mean (SD)		10.70 (10.65)		N/A	
Young Mania Rating Scale total, mean (SD)		1.89 (4.35)		N/A	

^a^N/A: not applicable.

^b^Categories were not mutually exclusive.

^c^Missing for 1 patient.

^d^Missing for 29 patients.

^e^Missing for 55 patients and 3 controls.

We also have one study that responds to NIMH Strategic Aim 2.2 [12] to develop novel behavioral assessments to evaluate domains relevant to mental illness. This is a single-site study at UCSD (PI: author RCM, R21MH116104) with the goals of understanding the real-time effects of mood on real-world cognitive performance and discovering how real-world cognition relates to real-time daily functioning among individuals with bipolar disorder. Sixty-six participants aged 18-65 years have completed this study (36 with bipolar disorder I, 10 with bipolar disorder II, 20 controls; see Table 3 for baseline demographic and clinical characteristics of the sample), which included a baseline assessment followed by 14 days of smartphone-based EMA and mobile cognitive testing (administered 3 times per day for a total possibility of 42 EMAs per participant).

For the present study, we will follow up with these previously enrolled research participants to assess their current mental health and psychosocial functioning with the exact same questions that were utilized during their previous participation. This study design will allow us to directly compare participants’ prepandemic mental health functioning, based on dense sampling of their momentary responses regarding symptoms, functioning, and self-evaluations with mental health functioning during the acute phase of the COVID-19 pandemic. We will also be positioned to examine demographic, cognitive, and psychosocial factors that may be predictive of better and worse mental health outcomes in this unprecedented time. Therefore, the aims of this study are to learn about (1) the mental health impact of COVID-19 and social distancing behaviors among at-risk populations and (2) prevention behaviors taken to reduce the risk of COVID-19 infection among persons with SMI. In so doing, we will use a comprehensive and detailed set of previously collected EMA data (up to 90 observations per patient collected over a 30-day sampling period) and ask those same questions again in two telephone reassessments: the first round of telephone assessments will occur between April 3, 2020, and May 31, 2020; the second round will occur 1 month after reopening. Although this will differ by state, we anticipate a date between June 1, 2020, and August 31, 2020. Our results should provide vital information regarding the overall level of awareness individuals with SMI have regarding the health risks of COVID-19 and how it is currently impacting their daily lives.

**Table 2:** Participant demographic and clinical characteristics from parent study 2 (note: this study includes only individuals with a diagnoses of mental illness).

Characteristic		Suicidal ideation (n=48)	No suicidal ideation (n=49)
Sex (male), n (%)		22 (47)	23 (47)
**Race, n (%)**			
	Caucasian	14 (29)	17 (35)
	African American	17 (36)	28 (57)
	Native American	0 (0)	0 (0)
	Asian	3 (6)	1 (2)
	Native Hawaiian/Pacific Islander	0 (0)	1 (2)
	Other	14 (29)	2 (4)
**Ethnicity, n (%)**			
	Hispanic	16 (33)	7 (14)
	Non-Hispanic	32 (67)	42 (86)
**Diagnosis, n (%)**			
	Schizophrenia	14 (29)	23 (47)
	Schizoaffective disorder	24 (50)	18 (37)
	Bipolar disorder (with psychotic features)	8 (18)	7 (14)
	Major depressive disorder (without psychotic features)	1 (2)	1 (2)
**Employment status^a^, n (%)**			
	Employed, full time	2 (4)	0 (0)
	Employed, part time	6 (13)	7 (16)
	Unemployed	5 (12)	4 (9)
	Part-time student	1 (2)	0 (0)
	Full-time student	1 (2)	0 (0)
	Receiving disability	26 (59)	30 (68)
	Receiving disability, part-time work	2 (4)	3 (7)
	Retired	2 (4)	0 (0)
**Residential status^b^, n (%)**			
	Independent, financially responsible	31 (69)	30 (68)
	Independent, not financially responsible	10 (22)	11 (25)
	Residential facility, unsupervised	0 (0)	1 (2)
	Residential facility, supervised	4 (9)	2 (4)
Age (years)^c^, mean (SD)		44.04 (12.20)	44.6 (11.02)
Education (years)^d^, mean (SD)		12.40 (2.84)	12.81 (1.86)
Maternal education (years)^e^, mean (SD)		11.74 (4.13)	12.63 (3.44)
Paternal education (years)^f^, mean (SD)		13.83 (3.52)	13.14 (3.55)
Montgomery-Asberg Depression Rating Scale total^g^, mean (SD)		21.95 (11.2)	9.68 (9.86)
Young Mania Rating Scale total^h^, mean (SD)		2.30 (3.85)	1.21 (3.38)

^a^Not available (ie, data not entered prior to shelter-in-place orders and unavailable at this time) for 3 patients with suicide ideation and 5 patients without suicide ideation.

^b^Not available for 3 patients with suicide ideation and 5 patients without suicide ideation.

^c^Not available for 3 patients with suicide ideation and 5 patients without suicide ideation.

^d^Not available for 2 patients with suicide ideation and 5 patients without suicide ideation.

^e^Missing for 10 patients with suicide ideation and 11 patients without suicide ideation.

^f^Missing for 14 patients with suicide ideation and 16 patients without suicide ideation.

^g^Total not available for 4 patients with suicide ideation and 5 patients without suicide ideation.

^h^Total not available for 2 patients with suicide ideation and 5 patients without suicide ideation.

**Table 3:** Participant demographic and clinical characteristics for parent study 3.

Characteristic		Bipolar disorder (n=46)	Controls (n=20)
Sex (male), n (%)		16 (35)	6 (30)
**Race, n (%)**			
	Caucasian	26 (57)	8 (40)
	African American	4 (9)	2 (10)
	Asian	2 (4)	5 (25)
	Native Hawaiian/Pacific Islander	3 (7)	1 (5)
	Other	11 (24)	4 (20)
**Ethnicity^a^, n (%)**			
	Hispanic	8 (18)	2 (10)
	Non-Hispanic	37 (82)	18 (90)
**Diagnosis, n (%)**			
	Bipolar disorder I	14 (30)	N/A^b^
	Bipolar disorder II	10 (22)	N/A
	Bipolar disorder I (with psychotic features)	22 (48)	N/A
**Employment status, n (%)**			
	Employed, full time	13 (28)	12 (60)
	Employed, part time	4 (9)	4 (20)
	Unemployed	6 (13)	0 (0)
	Stay-at-home parent	0 (0)	0 (0)
	Part-time student	0 (0)	0 (0)
	Full-time student	1 (2)	1 (5)
	Receiving disability, unemployed	16 (35)	0 (0)
	Receiving disability, part-time work	5 (11)	1 (5)
	Retired	1 (2)	2 (10)
**Residential status, n (%)**			
	Independent, financially responsible	36 (78)	16 (80)
	Independent, not financially responsible	8 (17)	4 (20)
	Residential facility, unsupervised	1 (2)	0 (0)
	Residential facility, supervised	1 (2)	0 (0)
Age (years), mean (SD)		42.72 (11.42)	41.03 (14.56)
Education (years), mean (SD)		14.91 (2.52)	15.65 (2.74)
Maternal education (years)^c^, mean (SD)		14.20 (3.93)	12.83 (3.62)
Paternal education (years)^d^, mean (SD)		15.22 (3.04)	15.24 (3.07)
Montgomery-Asberg Depression Rating Scale total, mean (SD)		11.20 (8.50)	N/A
Young Mania Rating Scale total, mean (SD)		6.44 (5.64)	N/A

^a^Missing for 1 participant with bipolar disorder.

^b^N/A: not applicable.

^c^Missing for 1 participant with bipolar disorder and 2 controls.

^d^Missing for 9 participants with bipolar disorder and 3 controls.

## Methods

### Design

This study involves 2 telephone interviews during which participants will be readministered psychiatric symptom–related questions that they received during the parent study via EMA, with the major modification being that the questions for the present study will be administered via a telephone survey. These items are presented in Multimedia Appendix 1 as a combination of the three surveys (please note that each parent study had a slightly different EMA survey). Participants will also be asked new questions about how they are currently feeling, thinking about, and dealing with COVID-19. The survey will take approximately 30 minutes to complete.

### Study Population and Inclusion and Exclusion Criteria

All participants who are currently or have previously participated in one of our ongoing parent studies (N=352 participants to date; approximately 24% participants overlap between studies), and who consented to being contacted for future studies, will be called and invited to participate. In general, participants include adults between the ages of 18 and 65 years who have a diagnosis of schizophrenia, schizoaffective disorder, bipolar disorder (I or II), or major depression with psychotic features. All individuals are receiving only outpatient care and are free from neurological and/or neurodegenerative disorders. A small sample of psychiatrically healthy individuals is also included (n=35).

### Questionnaires

The parent study EMA questionnaires were developed by authors CAD, PDH, RCM, and AEP. All three questionnaires include items about engagement in daily activities and social interactions (Where are you? Who are you with? What are you doing?), mood (in the moment or since the past alarm), symptoms (eg, “since the past alarm, how often have you heard voices”), and other behavioral indicators of health (eg, sleep, substance use).

The newly developed COVID-19 exposure and prevention behavior questionnaire includes 16 items on exposure and prevention behaviors (Multimedia Appendix 2). We will also be administering open-source scales to assess the psychological impacts of COVID-19, including the Center for Epidemiologic Studies Depression Scale [13], National Institutes of Health PROMIS (Patient-Reported Outcomes Measurement Information System) emotional distress-anxiety scale [14], Perceived Stress Scale [15], 3 items from the UCLA Loneliness Scale [16], modified to be specific to COVID-19, the 6-item Lifetime Orientation Test-Revised (LOT-R; measure of optimism) [17], Satisfaction with Life Scale [18], Duke Social Support Scale-Social Interaction Subscale (4 items) [19], and an 11-item brief coping scale [20]. The corresponding author can be contacted to request a complete packet of these measures.

#### Consent

This study was approved by each participating university’s Institutional Review Board. Participants will provide verbal consent on the phone and will be compensated for their participation.

### Data Analysis Plan

The estimated sample size is 200. For aim 1, the primary outcome will be change in average mood ratings (sadness, relaxed, energized, happiness, anxious) from the previous EMA surveys to now (spring 2020, when shelter-in-place orders are effective), then again during the summer of 2020 (unknown if shelter-in-place orders will be effective or if people have returned to a “normal” life). Changes in these outcomes will be evaluated using a mixed-models repeated measures analysis of variance with restricted maximum likelihood estimation. Group membership (schizophrenia, schizoaffective disorder, bipolar disorder) and assessment point (baseline, follow-up) will be treated as fixed effects and participants will be treated as a random effect. The group-by-time interaction will be the fixed effect of interest. Secondary analyses will be conducted to evaluate (1) the predictors of change from baseline, with a focus on diagnosis and psychotic symptoms, examined with regression models, and (2) group differences at each time point and differences in change scores between controls (n=35) and patient groups.

For aim 2, the primary outcome will be characterization of prevention behavior by group. For studies and participants where we have this information, we will also relate these data to assessments of insight, including clinical insight, self-monitoring ability collected during EMA, and the results of a comprehensive assessment of the ability to evaluate one’s own performance on an array of neurocognitive, social cognitive, and functional measures. These analyses will be examined with correlational statistics, including regression models.

## Results

The inclusion of participants for the first round of telephone assessments started on April 3, 2020 and will be completed by May 31, 2020. As of April 30, 2020, 101 individuals had completed these first-round assessments. The second round of telephone assessments will likely occur between June 1, 2020, and August 31, 2020. Study results will be published in peer-reviewed scientific journals in a timely fashion at completion of data collection. Data addressing non-COVID-19 topics from the sample collected to date are already being submitted for publication to scientific journals.

## Discussion

### Principal Findings

This study will shed light on the direct impact of the COVID-19 pandemic on the well-being of people with serious mental illness, a largely overlooked yet vulnerable population during this pandemic. Individuals with mental illness are often burdened not only by their illness but also by social isolation, under- or unemployment, lower socioeconomic status, cognitive impairments, and limited access care. Such individuals may therefore represent a particularly vulnerable and important group in whom we must strive to understand the effects of COVID-19. Findings from this study have the potential to characterize the degree of distress among persons with SMI during this pandemic and will also help to clarify whether individuals with SMI are able to protect themselves and others from infection. These findings can also help us identify risk and resiliency factors predictive of positive and negative outcomes to this high-stress situation, which could provide targets for early intervention in the (likely inevitable) event that another pandemic occurs and/or that social distancing measures are necessary in the future. Lastly, we hope this protocol paper will provide a roadmap and resources for other researchers who would like to implement a similar approach in their studies.

## Appendix

Multimedia Appendix 1 Psychiatric symptom EMA questions from parent studies (combined), adapted for a telephone follow-up assessment. Includes skip-logic.

Multimedia Appendix 2 COVID-19 exposure and prevention behavior questionnaire.

## Abbreviations

COVID-19: coronavirus disease 2019
EMA: ecological momentary assessment
NIMH: National Institute of Mental Health
PI: principal investigator
SMI: serious mental illness
