# Supplementary material for: Assessing the Real-Time Mental Health Challenges of COVID-19 in Individuals With Serious Mental Illnesses: Protocol for a Quantitative Study
Source: JMIR Res Protoc. 2020 May 22;9(5):e19203. doi: 10.2196/19203 (PMC7247464; doi:10.2196/19203)
Supplement: Multimedia Appendix 1 [file resprot_v9i5e19203_app1.docx]

**Multimedia 1. Psychiatric Symptom EMA Questions from Parent Studies (Combined), Adapted for a Telephone Follow-Up Assessment. Includes skip-logic.**

1. What time did you attempt to go to sleep last night? *(Range: 7:00pm-7:30pm to 3:30am-4:00am; Other)*
2. How many hours of sleep did you get? *(Rater note: can be on the hour, or can include 10, 20, 30, 40, or 50 minutes from 0-14hr)*
3. How restful was your sleep? *(1 – Not at all restful to 7 – Very restful)*

___________________________________________________________________

1. *Where are you right now? ___________________________________________________________________
2. Who is with you at the moment? *(Rater note: Can choose multiple) (Alone; Spouse or partner; Friends; Other family members; With healthcare provider; With other known people; With unknown people)*
3. Yesterday, how many times did you have a brief (<15 minutes) video of phone interaction with someone? (e.g., talking on phone, Facetime, Zoom)? *(integer or x+ e.g.: 4+)*
4. Yesterday, how many times did you have a long (>15 minutes) video interaction with someone? (e.g., talking on phone, Facetime, Zoom)? *(integer or x+ e.g.: 4+)*
5. Yesterday, how many times did you have a brief (<30 minutes) non-verbal interaction with someone? (e.g., text, written chat) today? *(integer or x+ e.g.: 4+)*
6. Yesterday, how many times did you have a long (>30 min) non-verbal interaction with someone? (e.g., text, written chat) today? *(integer or x+ e.g.: 4+)*
7. **What are you doing? *(Rater note: can state more than one answer, if pt marks “at home” for question #4 go to options 7a, otherwise 7b)*
8. How did you get to where you are? *(Rater note: not answered if pt. marked “at home” for question # 4) Walked; Got a ride from someone; Took public transportation; Drove myself; Other)*

____________________________________________________________________

1. How positive were your feelings yesterday on a scale of 1 - 7? *(1 being “Not at all”, and 7 being “Extremely”)*
2. *Since yesterday, who have you interacted with? (*I haven’t interacted with others; Friends; Spouse, partner or other family; With healthcare provider; With strangers; With roommates, coworkers, or others you know)*
3. In the last 3-4 hours, who have you interacted with? *(I haven’t interacted with others; Friends; Spouse, partner or other family; With healthcare provider; With strangers; With roommates, coworkers, or others you know)*

*If no interaction on #13 (since yesterday):*

1. How much interest or motivation did you have interacting with others since yesterday on a scale of 1 - 7? *(1 being “Not at all”, and 7 being “Very much”)*

*If no interaction on #14 or #13:*

1. How much interest or motivation did you have interacting with others in the past 3-4 hours on a scale of 1 – 7? *(1 being “Not at all”, and 7 being “Very much”)*

Since yesterday *(only answer if yes interaction on #13):*

1. How much pleasure or enjoyment did you feel in these interactions yesterday on a scale of 1 - 7? *(1 being “Not at all”, and 7 being “Very much”)*
2. How did you feel toward others in these interactions yesterday on a scale of 1 – 7? *(1 being “On guard or threatened, and 7 being “Trusting or Warm”)*
3. What do you think others were thinking about you in yesterday’s interactions on a scale of 1 - 7? *(1 being “Unlikeable or inferior, and 7 being Likable or capable)*

In the past 3-4 hours *(only answer if yes interaction on #14):*

1. How much pleasure or enjoyment did you feel in these interactions in the past 3-4 hours on a scale of 1 - 7? *(1 being “Not at all”, and 7 being “Very much”)*
2. How did you feel toward others in these interactions in the past 3-4 hours on a scale of 1 – 7? *(1 being “On guard or threatened, and 7 being “Trusting or Warm”)*
3. What do you think others were thinking about you in the interactions in the past 3-4 hours on a scale of 1 - 7? *(1 being “Unlikeable or inferior, and 7 being Likable or capable)*
4. How engaged with other people were you yesterday on a scale of 1 - 7? *(1 being “Not at all”, and 7 being “Extremely”)*
5. How well were you able to accomplish your goals yesterday on a scale of 1 – 7? *(1 being “Not at all”, and 7 being “Extremely”)*
6. How confident did you feel yesterday on a scale of 1 - 7? *(1 being “Not at all”, and 7 being “Extremely”)*
7. How meaningful were your activities yesterday on a scale of 1 - 7? *(1 being “Not at all”, and 7 being “Extremely”)*

Current Emotions:

1. What is your current mood? *(Most ever manic; Severe mania; Moderate mania; Mild mania (hypomania); Euthymic; Mild depression; Moderate depression; Severe depression; Most ever depressed)*
2. Are you feeling tired on a scale of 1 - 7? *(1 being “Not at all”, and 7 being “Extremely”)*
3. Are you feeling relaxed on a scale of 1 – 7?*(1 being “Not at all”, and 7 being “Extremely”)*
4. Are you feeling happy on a scale of 1 - 7? *(1 being “Not at all”, and 7 being “Extremely”)*
5. Are you feeling energized or excited on a scale of 1 – 7?*(1 being “Not at all”, and 7 being “Extremely”)*
6. Are you feeling sad or depressed on a scale of 1 – 7? *(1 being “Not at all”, and 7 being “Extremely”)*
7. Are you feeling anxious or nervous on a scale of 1 -7? *(1 being “Not at all”, and 7 being “Extremely”)*
8. Are you feeling angry or upset on a scale of 1 - 7? *(1 being “Not at all”, and 7 being “Extremely”)*
9. Are you feeling stressed on a scale of 1 - 7? *(1 being “Not at all”, and 7 being “Extremely”)*
10. Are you feeling impulsive on a scale of 1 – 7? *(1 being “Not at all”, and 7 being “Extremely”)*
11. Are you feeling forgetful on a scale of 1 - 7? *(1 being “Not at all”, and 7 being “Extremely”)*
12. Are you having difficulty in concentrating on a scale of 1-7? *(1 being “Not at all”, and 7 being “Extremely”)*

Past 3-4 hours:

1. In the past 3-4 hours, how much have you felt sad or depressed on a scale of 1 – 7? *(1 being “Not at all”, and 7 being “Extremely”)*
2. In the past 3 – 4 hours, how much have you felt energized or excited on a scale of 1 - 7? *(1 being “Not at all”, and 7 being “Extremely”)*
3. In the past 3 – 4 hours, how much have you been feeling like you belong or fit with others in your life on a scale of 1 - 7? *(1 being “Not at all”, and 7 being “Extremely”)*
4. In the past 3 – 4 hours, how much have felt irritated or upset on a scale of 1 - 7? *(1 being “Not at all”, and 7 being “Extremely”)*
5. In the past 3 – 4 hours, how much have you felt that you were a burden on others on a scale of 1 - 7? *(1 being “Not at all”, and 7 being “Extremely”)*
6. In the past 3 – 4 hours, how much have you felt happy on a scale of 1 – 7? *(1 being “Not at all”, and 7 being “Extremely”)*
7. In the past 3 – 4 hours, how much have you been bothered by voices on a scale of 1 – 7? *(1 being “Not at all”, and 7 being “Extremely”)*
8. In the past 3 – 4 hours, how much have you had thoughts that you really can’t trust other people on a scale of 1 -7? *(1 being “Not at all”, and 7 being “Extremely”)*

Concluding Questions:

1. Yesterday, do you think you were successful in completing your goals for the day? *(Not at all; A little bit; Somewhat; Quite a bit; Very much*
2. Are you satisfied with how you spent your day yesterday? *(Not at all; A little bit; Somewhat; Quite a bit; Very much*
3. ***If you could redo yesterday, what would you do differently? *(Rater note: can choose more than one option)*

___ Nothing differently

___ Get more exercise

___ Do more chores/errands

___ Spend more time socializing

___ Spend more time at home

___ Spend less time at home

___ Watch more TV

___ Get more rest

___ Spend more time in mentally stimulating activities (e.g., working, reading)

1. Since yesterday, how much have you been feeling like you belong or fit with others in your life on a scale of 1 - 7? *(1 being “Not at all”, and 7 being “Extremely”)*
2. Since yesterday, how much have you felt that you were a burden on others on a scale of 1 - 7? *(1 being “Not at all”, and 7 being “Extremely”)*
3. Since yesterday, how much have you been bothered by voices of 1 – 7? *(1 being “Not at all”, and 7 being “Extremely”)*
4. Since yesterday, how much have you had thoughts that you really can’t trust other people on a scale of 1 -7? *(1 being “Not at all”, and 7 being “Extremely”)*
5. How much interest or motivation do you have in interacting with others later today on a scale of 1 - 7? *(1 being “Not at all”, and 7 being “Very much”)*
6. How much do you want to avoid others later today on a scale of 1-7? *(1 being “Not at all”, and 7 being “Very much”)*
7. ****Today, have you taken or used any of the following substances? *(Rater note: can select more than one answer)*

*___ Caffeine*

*___ Tobacco*

*___ Herbal supplements*

*___ Weight-loss supplements*

*___ Alcohol*

*___ Cannabis/marijuana*

*___ Cocaine/crack*

*___ Crystal/meth*

*___ Ecstasy/Molly*

*___ Heroin*

*___ Other street drug(s)*

*___ Prescription drugs not prescribed to me*

*___ No substance/drug use*

1. In thinking about your day yesterday, what was your level of well-being on a scale of 1-7? *(1 being “Very low”, and 7 being “Very high”)*
2. In thinking about your day today, what is level of well-being on a scale of 1-7? *(1 being “Very low”, and 7 being “Very high”)*
