# Supplementary material for: Assessing the Real-Time Mental Health Challenges of COVID-19 in Individuals With Serious Mental Illnesses: Protocol for a Quantitative Study
Source: JMIR Res Protoc. 2020 May 22;9(5):e19203. doi: 10.2196/19203 (PMC7247464; doi:10.2196/19203)
Supplement: Multimedia Appendix 2 [file resprot_v9i5e19203_app2.docx]

**Multimedia Appendix 2. COVID-19 Exposure and Prevention Behavior Questionnaire**

|  |  | YES | NO |
| --- | --- | --- | --- |
| 1. | Have you been diagnosed with COVID-19 | 1 | 2 |
| 2. | Have you been exposed to someone likely to have COVID-19 | 1 | 2 |
| 3. | Has anyone in your family been diagnosed with COVID-19? | 1 | 2 |
| 4. | Have you been suspected of having Coronavirus/COVID-19 infection?   - 1. Yes, had positive test   2. Yes, waiting for a test result   3. Yes, have had some possible symptoms, but no test given   4. Yes, I believe I am infected, but I do not have symptoms   5. No symptoms or signs | | |
| 5. | Have any of the following happened to your family members because of COVID-19? (check all that apply)   - - - - 1. Fallen ill physically         2. Hospitalized         3. Put into self-quarantine with symptoms         4. Put into self-quarantine without symptoms (e.g., due to possible exposure)         5. Lost job   1. Reduced ability to earn money   2. Passed away   3. None of the above | | |
| 6. | In the last month, have you: | | |
| 7. | Self-distanced (chosen to stay at home and avoid contact with other people)? | 1 | 2 |
| 8. | Worked or volunteered remotely? | 1 | 2 |
| 9. | Avoided public spaces or public transportation? | 1 | 2 |
| 10. | Avoided seeing friends and family members in person? | 1 | 2 |
| 11. | Stayed at home except for essential reasons (food, gas, medical) | 1 | 2 |
| 12. | Cleaned hands by handwashing or sanitizer? | 1 | 2 |
| 13. | Used a facemask/scarf? | 1 | 2 |
| 14. | Worn gloves? |  |  |
| 15. | Cleaned or disinfected frequently touched surfaces? | 1 | 2 |
| 16. | Have the restrictions on leaving home prevent you from seeking health care/contacting a health care provider or seeking counseling/therapy? | 1 | 2 |
| 16a. | If yes, why? (check all that apply)   - 1. Fear of being infected with Coronavirus in a health care facility   2. Fear of infecting someone else with Coronavirus upon visiting a health care facility   3. Tried to seek care, but was turned away   4. My usual care provider is not available due to the COVID19/Coronavirus crisis, and I do not know where else to go   5. Other: ___________________________ | | |
